# Hemolytic Dynamics of Weekly Primaquine Antirelapse Therapy Among Cambodians With Acute *Plasmodium vivax* Malaria With or Without Glucose-6-Phosphate Dehydrogenase Deficiency

**DOI:** 10.1093/infdis/jiz313

**Published:** 2019-09-24

**Authors:** Walter R J Taylor, Sim Kheng, Sinoun Muth, Pety Tor, Saorin Kim, Steven Bjorge, Narann Topps, Khem Kosal, Khon Sothea, Phum Souy, Chuor Meng Char, Chan Vanna, Po Ly, Virak Khieu, Eva Christophel, Alexandra Kerleguer, Antonella Pantaleo, Mavuto Mukaka, Didier Menard, J Kevin Baird

**Affiliations:** 1 National Center for Parasitology, Entomology, and Malaria Control, Phnom Penh, Cambodia; 2 Institut Pasteur du Cambodge, Phnom Penh, Cambodia; 3 World Health Organization (WHO) Cambodia Country Office, Phnom Penh, Cambodia; 4 Pailin Referral Hospital, Pailin, Cambodia; 5 Anlong Veng Referral Hospital, Anlong Venh, Cambodia; 6 Pramoy Health Center, Veal Veng, Cambodia; 7 Service de Médecine Tropicale et Humanitaire, Hôpitaux Universitaires de Genève, Switzerland; 8 Mahidol Oxford Tropical Medicine Research Unit, Bangkok, Thailand; 9 WHO Western Pacific Regional Office, Manila, the Philippines; 10 Department of Biomedical Science, University of Sassari, Italy; 11 Centre for Tropical Medicine, Nuffield Department of Medicine, University of Oxford, United Kingdom; 12 Malaria Genetics and Resistance Group, Biology of Host-Parasite Interactions Unit, Institut Pasteur, Paris, France; 13 Eijkman Oxford Clinical Research Unit, Eijkman Institute of Molecular Biology, Jakarta, Indonesia

**Keywords:** Primaquine, glucose-6-phosphate dehydrogenase deficiency, malaria, hemoglobin E, Cambodia

## Abstract

**Background:**

Hemoglobin (Hb) data are limited in Southeast Asian glucose-6-phosphate dehydrogenase (G6PD) deficient (G6PD^−^) patients treated weekly with the World Health Organization–recommended primaquine regimen (ie, 0.75 mg/kg/week for 8 weeks [PQ _0.75_]).

**Methods:**

We treated Cambodians who had acute *Plasmodium vivax* infection with PQ_0.75_ and a 3-day course of dihydroartemisinin/piperaquine and determined the Hb level, reticulocyte count, G6PD genotype, and Hb type.

**Results:**

Seventy-five patients (male sex, 63) aged 5–63 years (median, 24 years) were enrolled. Eighteen were G6PD deficient (including 17 with G6PD Viangchan) and 57 were not G6PD deficient; 26 had HbE (of whom 25 were heterozygous), and 6 had α-/β-thalassemia. Mean Hb concentrations at baseline (ie, day 0) were similar between G6PD deficient and G6PD normal patients (12.9 g/dL [range, 9‒16.3 g/dL] and 13.26 g/dL [range, 9.6‒16 g/dL], respectively; *P* = .46). G6PD deficiency (*P* = <.001), higher Hb concentration at baseline (*P* = <.001), higher parasitemia level at baseline (*P* = .02), and thalassemia (*P* = .027) influenced the initial decrease in Hb level, calculated as the nadir level minus the baseline level (range, −5.8–0 g/dL; mean, −1.88 g/dL). By day 14, the mean difference from the day 7 level (calculated as the day 14 level minus the day 7 level) was 0.03 g/dL (range, −0.25‒0.32 g/dL). Reticulocyte counts decreased from days 1 to 3, peaking on day 7 (in the G6PD normal group) and day 14 (in the G6PD deficient group); reticulocytemia at baseline (*P* = .001), G6PD deficiency (*P* = <.001), and female sex (*P* = .034) correlated with higher counts. One symptomatic, G6PD-deficient, anemic male patient was transfused on day 4.

**Conclusions:**

The first PQ_0.75_ exposure was associated with the greatest decrease in Hb level and 1 blood transfusion, followed by clinically insignificant decreases in Hb levels. PQ_0.75_ requires monitoring during the week after treatment. Safer antirelapse regimens are needed in Southeast Asia.

**Clinical Trials Registration:**

ACTRN12613000003774.

## Teaser

Among *Plasmodium vivax*–infected Cambodians with glucose-6-phopshate dehydrogenase (G6PD) deficiency who were treated weekly with primaquine, 6 had marked decreases in the hemoglobin (Hb) level, and 1 was transfused. G6PD deficiency and HbE were consistently important factors associated with changes in the Hb level over time.

The *Plasmodium vivax* life cycle includes blood-stage parasites that cause acute febrile illnesses and latent liver hypnozoites that activate over variable periods, leading to renewed clinical attacks, called “relapses.” The natural frequency of relapse due to Southeast Asian tropical *P. vivax*, like that for the Chesson strain, is high, with a median incidence density of 5–6 attacks/person-year following primary infection [1]. Relapses are important because they account for >80% of the prevalent patency of *P. vivax* in Thailand [2] and Papua New Guinea [3] and are associated with significant morbidity and mortality [4, 5].

Currently, the 8-aminoquinolines primaquine (PQ) and tafenoquine (TQ) are the only available drugs that kill hypnozoites and prevent relapses. Tropical vivax strains are tolerant of PQ and require treatment with 0.5 mg/kg body weight/day for 14 days (7 mg/kg total) to achieve good efficacy [6, 7]. PQ and TQ provoke dose-dependent acute hemolytic anemia in patients with glucose-6-phosphate dehydrogenase (G6PD) deficiency, an X-linked recessive, red blood cell (RBC) enzymopathy whose variants have differing degrees of sensitivity to 8-aminoquinolines [8–11]. G6PD catalyzes the rate-limiting reaction in the hexose monophosphate shunt and serves to stabilize reserves of reduced glutathione in the RBC cytosol. A redox equilibrium favoring oxidized species of 5-hydroxylated metabolites of PQ may lead to irreversible hemochrome ligands that precipitate as Heinz bodies and lead to intravascular and extravascular hemolysis [12–14].

Daily delivery of PQ to G6PD deficient (G6PD^−^) individuals may result in potentially life-threatening acute hemolytic anemia [15], but work in the 1950s found that weekly PQ dosed at 0.75 mg/kg/week for 8 weeks (PQ_0.75_) caused considerably less hemolysis in G6PD^−^ African Americans (presumed to have mild G6PD deficiency due to G6PD A^−^) who were experimentally infected with Chesson strain *P*. *vivax*. Decreases in the hemoglobin (Hb) level relative to baseline were 7% during weekly PQ therapy and 20% and 50% with 15 mg and 30 mg, respectively, of daily PQ therapy [16]. When used in patients with the Viangchan variant, which confers moderately severe G6PD deficiency, PQ_0.75_ caused a 4-fold higher absolute decrease (*P* = .0002) in the median Hb level between baseline and day 7, compared with *P. vivax*–infected G6PD normal (G6PD^+^) patients (−2.2 g/dL [interquartile range {IQR}, −4.9‒0.8 g/dL] vs −0.5 g/dL [IQR, −2.2‒2.8 g/dL]). Moreover, 1 G6PD^−^ male patient developed symptomatic anemia (Hb level, 7.2 g/dL; baseline Hb level, 10 g/dL), necessitating a blood transfusion [17].

Patients with malaria who are treated with antimalarial drugs other than PQ or TQ also experience hematological changes. In uncomplicated falciparum malaria, mean reticulocyte counts decrease during the first 3 days and increase after parasite clearance, peaking usually on day 7 [18, 19] but as late as day 21 [20]; limited data from patients with acute *P. vivax* infection are consistent [17]. In falciparum and vivax malaria, the mean Hb level decreases initially, recovers, and stabilizes to normal levels after 4–6 weeks [21–23].

We have reported on the safety and tolerability of PQ_0.75_ in *P. vivax*–infected G6PD^−^ and G6PD^+^ Cambodians [17]. Herein, we model those Hb responses and other markers of hemolysis and explore their determinants.

## MATERIALS AND METHODS

### Trial Design, Study Site and Conduct, and Ethical Approval

Trial details are described elsewhere [17]. Briefly, from January 2013 to January 2014, 75 nonpregnant Cambodians aged >1 year with uncomplicated *P*. *vivax* malaria were treated with dihydroartemisinin/piperaquine (dihydroartemisinin target dose, 2 mg/kg/day) on days 0, 1, and 2 and PQ_0.75_ during days 0‒49, with individuals weighing 10‒17 kg receiving 7.5 mg of PQ weekly, those weighing 10‒25 kg receiving 15 mg, those weighing 26‒35 kg receiving 22.5 mg, those weighing 36‒45 kg receiving 30 mg, those weighing 46‒55 kg receiving 37.5 mg, those weighing 56‒75 kg receiving 45 mg, and those weighing ≥76 kg receiving 60 mg. Recruitment took place in Pailin (on the border between Thailand and Cambodia), Anlong Venh (in northwestern Cambodia), and Veal Veng (in western Cambodia).

Laboratory investigations included Giemsa-stained thick and thin blood films (*P. vivax* parasitemia was determined by counting the number of parasites/200 white blood cells on a thick blood film, assuming a total white blood cell count of 8000 cells/µL); thin blood films for detection of reticulocytemia; measurement of the Hb concentration (HemoCue, Ängelholm, Sweden); electrophoretic analysis of Hb, using the Minicap System (Sebia, Norcross, France) [24]; determination of G6PD genotype by polymerase chain reaction [24]; quantification of G6PD enzyme activity, using the Trinity Biotech (Ireland) quantitative G6PD assay, adapted for use on the Integra 400 analyzer (Roche Diagnostics, Meylan, France); a full blood count on days 0, 7, 28, and 56, using the CellDyn 3200 analyzer (Abbott, Rungis, France); and routine biochemistry analysis and measurement of the haptoglobin and lactate dehydrogenase (LDH) levels. The urine color was ranked using the Hillmen color chart (scale, 1–10) [25]; when considered paler than the color assigned a score of 1, the urine color was scored as 0.

Ethical approval was obtained from the National Ethical Committee for Health Research of the Cambodian Ministry of Health (Phnom Penh; reference no. 225 NECHR) and the ethical review board of the Western Pacific Regional Office of the World Health Organization (Manila, the Philippines; reference no. 2011. 08. CAM. 01. MVP). The study is registered with the Australian New Zealand Clinical Trials Registry (registration no. ACTRN12613000003774; 3 January 2013).

### Definitions

The absolute change in Hb level and the fractional change in Hb level in individual patients (ie, not the population nadir level) were determined, with the former calculated as [nadir Hb level] − [day 0 Hb level] and the latter calculated as 100 × [(change in absolute Hb level)/(day 0 Hb level)]. The total malaria-attributable change in Hb level following treatment was calculated as [day 56 Hb level] − [nadir Hb level]. For patients without day 56 Hb concentrations, the nadir Hb level was determined if there was a decrease followed by an increase in Hb concentration. Recovery of the Hb level in a given patient was defined as a day 56 Hb concentration greater than the baseline concentration. We defined a clinically concerning decrease in Hb level as an absolute decrease of >3 g/dL and/or a fractional decrease of >25%. The decrease in the uninfected red blood cell count per patient was calculated as [total decrease in the RBC count] – [decrease in the infected RBC count] (Supplementary Materials).

### Data Management and Statistical Methods

Data were double entered into Epidata, checked, and analyzed using Stata v14 (Stata, College Station, TX). Proportional data between groups were compared using χ ^2^ analysis or the Fisher exact test, as appropriate, and those within groups were analyzed using the Maentel Haenszel test and the exact McNemar significance probability. Continuous data were analyzed by paired (within-group) or unpaired (between-group) *t* tests for normally distributed data; corresponding nonparametric tests were the Wilcoxon signed-rank test and the Mann-Whitney *U* test.

We used multiple linear regression (a backward stepwise approach) to determine factors (eg, age, sex, baseline Hb level, G6PD status, Hb type, baseline parasitemia level, and length of illness) associated with the baseline Hb level, the absolute change in the Hb level, the total malaria-attributable change in the Hb level following treatment, and the decrease in RBC count. A linear mixed-effects regression model was used to determine factors associated with changes over time in the Hb level, reticulocytemia, the creatinine level, the LDH level, the haptoglobin level, and the conjugated and unconjugated bilirubin levels. Logistic regression was performed to assess factors associated with recovery of the Hb level and a clinically concerning decrease in the Hb level.

## RESULTS

### Patient Disposition and Baseline Characteristics

We recruited 75 acutely ill patients with microscopy-confirmed *P. vivax* monoinfection; most (80%) were young men in their 20s, and 15 (20%) were aged 5‒17 years (Table 1). Sixty-seven completed the trial to day 56, and none had recurrent malaria parasitemia (Supplementary Materials).

**Table 1.** Baseline Characteristics as a Function of Glucose-6-Phosphate Dehydrogenase (G6PD) Status

Parameter	G6PD Normal (n = 57)	G6PD Deficient (n = 18)	*P*
Age			
Overall, y	26.5 (7–63)	26.9 (5–56)	.88
<18 y	10 (17.5)	5 (27.8)	.34
Male sex	48 (84.2)	15 (83.3)	.93
Weight, kg	52.0 (14–88)	50.4 (20–56)	.63
Days ill, no.	3 (0–13)	2.4 (0–8)	.29
Primaquine dose, mg/kg			
Median (IQR)	0.73 (0.69–0.77)	0.74 (0.6–90.75)	.37
Range	0.53–0.98	0.65–0.78	
Hematologic			
Hb level			
Overall, g/dL	13.26 (91–6.3)	12.94 (9.6–16)	.48
Normal	31 (54.4)	11/17 (64.7)	
HbE genotype			
Heterozygous	20 (35.1)	5/17 (29.4)	
Homozygous	1 (1.75)	0	
α-thalassemia	1 (1.75)	1/17 (5.9)	.60
β-thalassemia	4 (7.1)	0	
G6PD activity			
Overall, U/g Hb	11.9 (6.9–18.5)	0.88 (0.1–1.5)^a^	<.001
Percentage of normal population median	99.2 (57.5–154.2)	7.3 (0.8–12.5)^a^	
Anemia^b^	21 (36.8)	6 (33.3)	1.0
Reticulocyte count, % of RBCs	1.5 (0.5–4.5)	1.86 (0.6–3.8)	.10
Biochemical			
Unconjugated bilirubin level			
Overall, mg/L	5.4 (0.8–14.6)	6.5 (1.4–15.3)	.22
High (≥0.8 mg/L)	9/56 (16.1)	6/17 (35.3)	.09
Conjugated bilirubin level			
Overall, mg/L	4.2 (0.5–14.3)	4.1 (0.6–9.8)	.96
High (≥2.0 mg/L)	47/56 (83.9)	12/17 (70.6)	.29
LDH level, IU/L	235 (23–611)	367 (127–800)	.0028
ALT level, IU/L	21.4 (4–149)	17.7 (9–36)	.46
Parasite			
*P. vivax* parasitemia, parasites/µL, median (range)	8300 (220–59 542)	6420 (159–9326)	.13

Data are mean (range) or no. or proportion (%) of patients, unless otherwise indicated.

Abbreviations: ALT, alanine aminotransferase; Hb, hemoglobin; IQR, interquartile range; LDH, lactate dehydrogenase; RBC, red blood cell.

^a^Two heterozygous female patients had baseline G6PD levels of 0.9 U/g Hb (7.9% of normal population median) and 1.3 U/g Hb (10.8% of normal population median).

^b^Hb concentrations were <13 g/dL and <12 g/dL in men and nonpregnant women (ages ≥15 y), respectively, and <12 g/dL and <11.5 g/dL in both sexes aged 12 to <15 years and 5 to <12 years, respectively.

Eighteen patients were G6PD deficient, of whom 15 were hemizygous males and 3 were heterozygous females; 17 and 1 had G6PD variants Viangchan and Canton, respectively. Female participants had significant lower baseline Hb concentrations, compared with males (12.1 vs 13.4 g/dL; *P* = .0009), and increasing age was significantly associated with a higher baseline Hb level (*P* = .001).

### Nadir Hb Concentration

Two patients had an immediate increase in the Hb level that was sustained to day 56. By day 7, 55 of 75 patients (73.3%) had reached their nadir Hb level (median time to nadir level, 3 days; interquartile range [IQR] 2–14 days; range, 0–49 days); day 2 was the most frequent day on which the nadir level was reached (21 of 74 patients [28.4%]; Supplementary Materials). The mean nadir Hb level was significantly lower in G6PD^−^ patients versus G6PD^+^ patients (10.32 g/dL vs 11.60 g/dL; *P* = .001). One symptomatic G6PD^−^ male patient was transfused after his Hb level decreased from 10 g/dL at baseline to 7.2 g/dL on day 4; posttransfusion Hb data were excluded from the analyses.

The mean absolute and fractional decreases in the Hb level were also significantly lower in the G6PD^−^ group, compared with the G6PD^+^ group, with changes in the absolute level of −2.61 g/dL and −1.65 g/dL, respectively (*P* = .001), and changes in the fractional level of −19.8% and −12.2%, respectively (*P* = .0001). Baseline Hb level, parasitemia level, G6PD deficiency, and thalassemia explained 45.4% (R^2^ = 45.4%) of the change in absolute Hb level (Table 2), and a higher level of G6PD enzyme activity was associated with a lower change in absolute Hb level (data not shown). Age, sex, length of illness, and PQ dose were not significant factors.

**Table 2.** Significant Explanatory Factors for Changes in Hemoglobin (Hb) Level, Reticulocyte Count, and Surrogate Biochemical Markers of Hemolysis

Parameter	Coefficient (95% CI)	*P*
Initial decrease in Hb level^a^		
G6PD deficiency	−1.26 (−1.73 to −.78)	<.001
Baseline Hb concentration	−0.37 (−.49 to −.25)	<.001
Baseline parasite count	−1.87 × 10^−5^ (−3.45 × 10^−5^ to −3.03 × 10^−6^)	.020
Thalassemia	−0.40 (−.80 to −.004)	.048
Uninfected RBC loss		
G6PD deficiency	1.17 × 10^6^ (.46 × 10^6^–1.87 × 10^6^)	.002
Baseline parasite count	27.8 (5.1–50.6)	.017
Thalassemia	654.1 × 10^3^ (74.1 × 10^3^–1.23 × 10^6^)	.028
Female sex	−916.4 × 10^3^ (−1.74 × 10^6^ to −90.8 × 10^3^)	.030
Age	27.1 × 10^3^ (1.5 × 10^3^–52.7 × 10^3^)	.038
Changes in Hb level over time		
Baseline Hb concentration	0.65 (.60–.70)	<.001
G6PD deficiency	−0.61 (−.79 to −.43)	<.001
Thalassemia	−0.47 (−.62 to −.32)	<.001
Female sex	−0.40 (−.64 to −.32)	<.001
Total malaria attributable decrease in Hb level		
G6PD deficiency	1.62 (1.03–2.21)	<.001
Primaquine level in mg/kg	5.19 (.65–9.74)	.026
Baseline parasite count	2.26 × 10^−5^ (1.57 × 10^−6^–4.36 × 10^−5^)	.036
	**Odds Ratio (95% CI)**	
Clinically concerning decrease in Hb level		
G6PD deficiency	12.6 (2.2–72.4)	.004
Baseline Hb level	1.93 (1.06–3.52)	.032
Good recovery in Hb level^b^		
Initial decrease in Hb level	4.4 (1.8–10.5)	.001
G6PD deficiency	22.7 (1.9–274.6)	.014
Baseline parasite count	1.00009 (1.00001–1.00016)	.021
	**Coefficient (95% CI)**	
Reticulocyte count		
Baseline reticulocyte count	0.29 (.21–.38)	.001
G6PD deficiency	0.33 (.13–.52)	<.001
Female sex	0.19 (.014–.38)	.034
Serum haptoglobin level		
G6PD deficiency	−0.21 (−.37 to −.05)	.010
Days of illness	−0.05 (−.076 to −.024)	<.001
Reticulocyte count dynamics	−0.087 (−.14 to −.026)	.005
Baseline parasite count	5.22 × 10^−6^ (5.17 × 10^−7^–9.93 × 10^−6^)	.030
Serum LDH concentration		
G6PD deficiency	115.9 (77.5–154.2)	<.001
Baseline temperature	−25.6 (−41.2 to −9.9)	.003
Serum unconjugated bilirubin concentration		
Temperature change over time	0.62 (.19–2.04)	.004
Change in Hb level over time	0.20 (.04–.37)	.012
Female sex	−0.71 (−1.41 to −.001)	.049

Abbreviations: CI, confidence interval; LDH, lactate dehydrogenase; RBC, red blood cell.

^a^Defined as [nadir Hb concentration] – [baseline concentration] in a given individual.

^b^This model excluded the primaquine level in mg/kg because it resulted in extreme odds ratios (and extreme 95% CIs) that were probably related to the small mg/kg range of 0.54–0.98.

### Clinically Concerning Decreases in Hb Level

The Hb level decreased by ≥5 g/dL in 2 patients and by >3 g/dL in 11 patients, and the level in 3 and 7 patients decreased fractionally by >30% and 25%, respectively, yielding 12 patients with a clinically concerning decrease in Hb level (Supplementary Materials). A higher baseline Hb level and G6PD deficiency were explanatory factors (Table 2).

### Changes in the Hb Level After the Second PQ Dose

Compared with day 7, the Hb level in 39, 30, and 3 patients had increased, decreased, and not changed, respectively, on day 14. Decreases in the Hb level were independent of G6PD status (4 of 17 [23.5%] in the G6PD^−^ group had a decrease, compared with 26 of 55 [47.3%] in the G6PD^+^ group; *P* = .099) and ranged from 0.3 to 1.8 g/dL (median decrease, 0.95 g/dL) in the G6PD^−^ group and from 0.1 to 3.2 g/dL (median decrease, 0.89 g/dL) in the G6PD^+^ group (*P* = .90). Of the 30 patients whose Hb level decreased, 17 reached their nadir Hb concentrations before day 7, and 15 had thalassemia (Supplementary Materials). The mean difference in Hb level between day 14 (12.54 g/dL) and day 7 (12.51 g/dL) was 0.03 g/dL (*P* = .80), compared with −0.69 g/dL between days 7 and 0 (*P* = .0001).

### Decreases in the RBC Count

The estimated total decrease in the RBC count from baseline to the day on which the nadir Hb level was achieved ranged from 0 to 5.41 × 10^6^ RBCs (IQR, 1.29 × 10^6^–3.40 × 10^6^ RBCs; median, 2.35 × 10^6^ RBCs); the decrease in the infected RBC count during this period was 522–260 496 RBCs (IQR, 12 592–48 776 RBCs; median, 26 301 RBCs). In patients whose RBC count decreased, the median ratio of the decrease in the uninfected RBC count to the decrease in the infected RBC count was 89.7:1 (IQR, 38:1–173:1) and was higher in G6PD^+^ patients (163:1) than in G6PD^−^ patients (70:1), but G6PD status was not statistically significant in a multivariate model (data not shown). G6PD deficiency, thalassemia, increasing age, baseline parasitemia level, and male sex enhanced the decrease in the uninfected RBC count (Table 2). No factors were significantly associated with a decrease in the infected RBC count, but there was a trend for an association with G6PD deficiency (*P* = .06).

### Changes in the Hb Level Over Time

**Figure 1.**
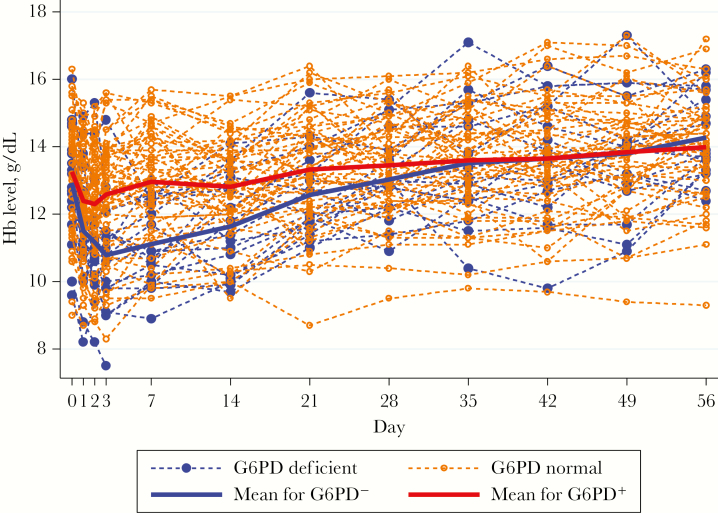
Hemoglobin (Hb) concentration (g/dL) changes over time as a function of G6PD status. The patient whose Hb level fell to <8 g/dL was transfused.

From days 0 to 56, Hb concentrations varied (Figure 1). The population nadir Hb level occurred on day 2 in the G6PD^+^ group and day 3 in the G6PD^−^ group. Thereafter, the G6PD^−^ group had a steeper mean increase in Hb level, approximating the level in the G6PD^+^ group by day 35. Significant negative factors influencing changes in the Hb level over time were female sex, G6PD deficiency, and thalassemia, whereas a higher baseline Hb level had a positive effect (Table 2).

For all patients (n = 67), the mean Hb level on day 56 was significantly higher than that at baseline (14.06 vs 13.22 g/dL; difference, 0.84 g/dL [IQR, 0.55–1.14 g/dL]; *P* < .0001). Overall, 52 patients (77.6%) achieved a recovery in the Hb level (median time of recovery, day 28; IQR, days 21–49), which was related to a greater absolute change in Hb level, G6PD deficiency, and a higher baseline parasitemia level (Table 2). The mean total malaria-attributable change in Hb level following treatment was 2.73 g/dL (range, 0.5–5.1 g/dL) and was higher in G6PD^−^ patients with a higher absolute change in Hb level and a higher baseline parasitemia level (Table 2).

### Reticulocyte Count Response

**Figure 2.**
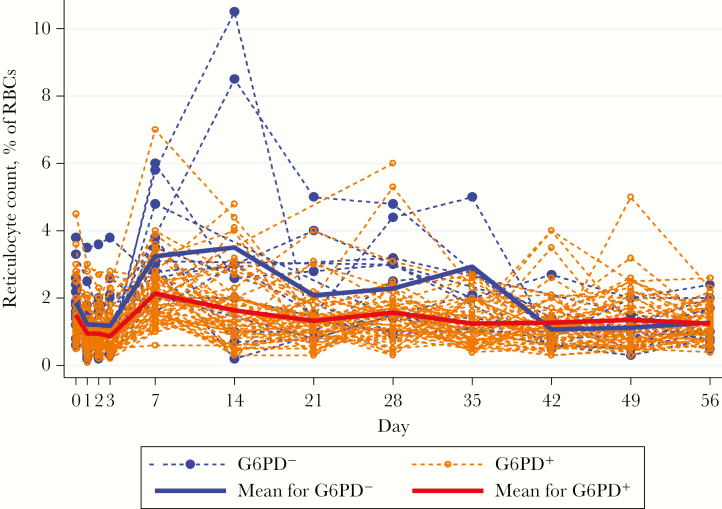
Reticulocytemia (%) over time, by G6PD status. RBC, red blood cell.

Mean reticulocyte counts decreased on days 1–3 and increased thereafter (Figure 2), with higher counts in G6PD^−^ patients, who experienced 2 peaks, on days 14 and 35; the reticulocyte counts in G6PD^+^ patients peaked once, on day 7. Baseline parasite count, G6PD status, and sex were explanatory factors (Table 2), but the absolute change in Hb level was not.

### Changes in Serum Haptoglobin, LDH, and Bilirubin Levels

**Figure 3.**
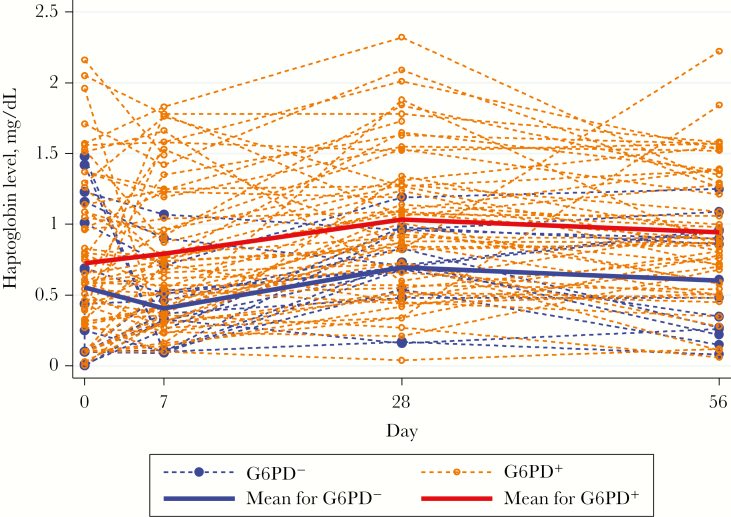
Changes in serum haptoglobin level (g/L) over time as a function of G6PD status.

**Figure 4.**
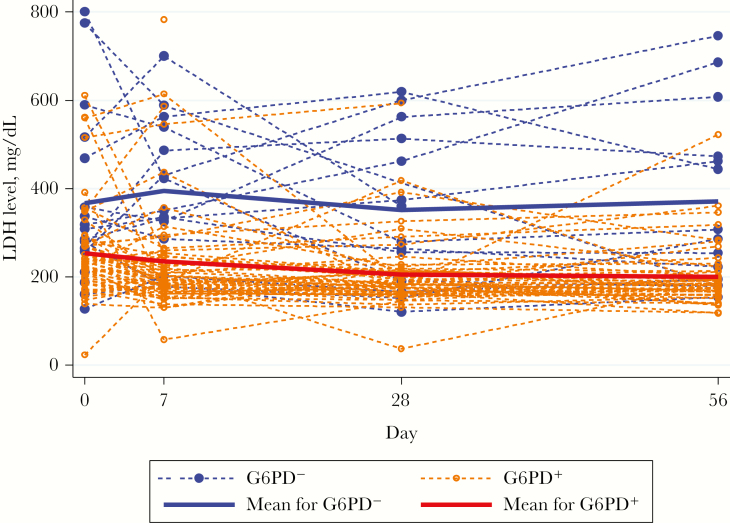
Changes in serum lactate dehydrogenase (LDH) concentrations (IU/L) over time, by G6PD status.

In G6PD^−^ patients, the mean serum haptoglobin concentration reach a nadir on day 7 and was significantly lower than that in G6PD^+^ patients (Figure 3). G6PD deficiency, length of illness, and reticulocyte count changes were inversely related and the day 0 parasitemia level directly related to changes in the haptoglobin level (Table 2). The mean serum LDH concentration rose initially in G6PD^−^ patients, peaking on day 7, and was significantly higher over time than that in the G6PD^+^ group (Figure 4 and Table 2).

The highest mean unconjugated and conjugated bilirubin concentrations were at baseline and decreased thereafter (Supplementary Materials). Sex, temperature, and changes in the Hb level explained changes in the unconjugated bilirubin level over time (Table 2), whereas only temperature was associated with higher conjugated bilirubin concentrations over time.

### Changes in the Serum Creatinine Level

**Figure 5.**
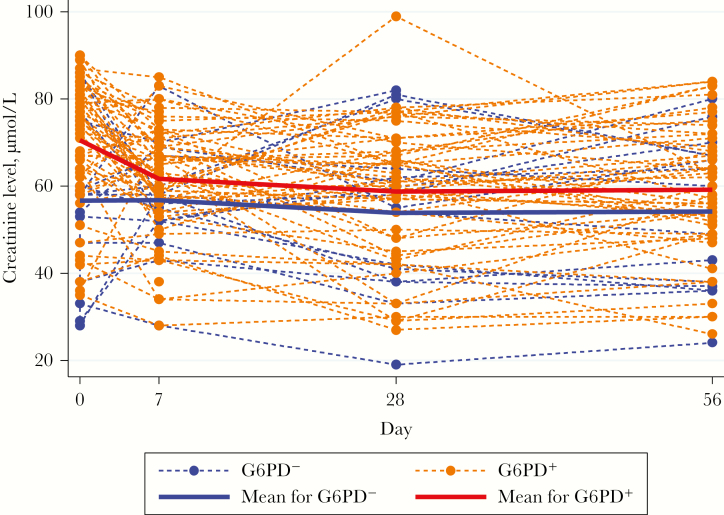
Serum creatinine concentrations (µmol/L) over time as a function of G6PD status.

Most creatinine concentrations were within normal limits, and mean values decreased over time, with a greater initial decrease in G6PD^+^ patients (Figure 5). On day 7, 21 of 71 patients (29.6%) had an increased creatinine level, including 5 patients with a clinically concerning decrease in the Hb level (Supplementary Materials), whereas the level in 50 (70.4%) had decreased (n = 47) or remained static (n = 3). No factors explained the change in creatinine level between days 0 and 7. Two patients had increases in the creatinine level within the first week, consistent with Kidney Disease Improving Global Outcomes criteria for stage 1 acute kidney injury [26]. The level in the male patient who was transfused increased by 50.1% (from 53 µmol/L at baseline to 79.6 µmol/L on day 5), and the level in an 11-year-old G6PD^−^ boy increased by 79.3% (from 29 µmol/L at baseline to 52 µmol/L on day 7).

### Changes in the Urine Color Over Time

The median Hillmen score at baseline was 3 in G6PD^−^ patients and 1 in G6PD^+^ patients, with a significantly different distribution between the groups (*P* = .009). Thereafter, maximum scores varied from 1 to 3 in the G6PD^−^ group and remained at 1 in the G6PD^+^ group (Supplementary Materials). The score for the transfused G6PD^−^ male patient increased from 0 and 4, whereas his Hb level decreased, with values of 10, 8.8, 8.2, 7.5, and 7.2 g/dL at baseline and on days 1, 2, 3, and 4, respectively (Supplementary Materials).

## DISCUSSION

This is the first study of World Health Organization–recommended weekly PQ therapy in Southeast Asia in which changes in the Hb level and other hemolytic markers over time are reported for patients with *P. vivax* malaria who have moderately severe G6PD deficiency or are G6PD normal. A minority of patients experienced marked decreases in the Hb level after the first PQ dose, including 1 patient who was transfused, but thereafter G6PD^−^ patients were tolerant of additional doses. G6PD status was the most consistent determinant of the degree of acute hemolytic anemia and recovery, whereas thalassemia and baseline parasite count were important contributors.

We reconfirm the greater posttreatment decrease in the uninfected RBC count as compared to infected RBCs (ratio, approximately 90 to 1), which is a higher figure than the ratio (34 to 1) reported in experimentally infected patients [27], and we identified factors pertinent only to the decreased uninfected RBC count. These findings support the important role of extravascular hemolysis as a major mechanism in the pathogenesis of anemia following treatment [28].

The contribution of thalassemia to the initial decrease in Hb level, through the enhanced decrease in the uninfected RBC count, is a new finding. HbE is very common in our region [29, 30] and, like β-thalassemia minor, is characterized by mild anemia and a rigid (ie, less deformable) and fragile RBC membrane; by contrast, α-thalassemic RBCs only have rigid membranes [31–33]. These membrane characteristics result in increased RBC destruction in the spleen.

A higher baseline parasite count was also associated with a greater initial decrease in Hb level and can be explained by a greater inflammatory response and oxidant environment [34], possibly enhancing physiological RBC destruction. Limited data suggest that intravascular hemolysis in *P. vivax* malaria is independent of the inflammatory response [35], consistent with the hypothesized dominance of extravascular hemolysis in hemolytic responses to therapy. Moreover, the observed low urine Hillmen scores support the relatively small contribution of intravascular hemolysis with weekly PQ dosing.

Our transfused patient developed darkening urine with a maximum Hillmen score of “only” 4 despite an almost 3-g/dL decrease in Hb level. Mildly dark urine may not arouse suspicion of anemia and could be confused with dehydration; hence, the critical need to ask about symptoms. Clinicians should be aware that the classic picture of pallor, jaundice, and cola-colored urine is seen typically with acute, massive hemolysis in favism and toxic doses of hemolytic drugs [36, 37]; thus, urine color may be an insensitive hemolytic marker at weekly therapeutic doses of PQ in sensitive patients. Nevertheless, prescribing clinicians should still warn patients about darkening urine, passing less urine, symptoms of anemia, and seeking early care.

Renal impairment is well described in favism and drug-induced acute hemolytic anemia and is usually reversible [38, 39]. The pathogenesis is incompletely defined but appears to be related to increased renal cellular uptake of free Hb that is converted to heme, resulting in oxidant damage to the tubules and glomeruli [40]. Most of our patients had normal creatinine concentrations, but 2 G6PD^−^ patients developed stage 1 acute kidney injury [26], which resolved without intervention. Reversible renal impairment was also seen in 30 Egyptian children aged 5 months to 7.5 years with acute hemolytic anemia (due to favism in 25), whose median estimated glomerular filtration rate increased from 73.6 mL/minute/1.73 m^2^ (range, 41.25‒125 mL/minute/1.73 m^2^) to 89.6 mL/minute/1.73 m^2^ (range, 82.5‒475 mL/minute/1.73 m^2^) 1 month after hemolysis [39].

The initial decrease in the Hb level to its nadir concentration was seen in approximately one half of patients by day 2 and in three quarters by day 7, and it paralleled decreases in mean reticulocyte counts, similar to *P. falciparum* infection [18]. Interestingly, a second PQ dose was associated with decreases in the Hb level in approximately 20% and 45% of G6PD^−^ and G6PD^+^ patients, respectively, including patients who had earlier reached their nadir Hb concentrations. Reassuringly, the limited decreases in the Hb level among G6PD^−^ patients and the overall mean increase in their Hb level between the first and second PQ dose suggest a degree of tolerance to additional oxidant stress from PQ. We hypothesize that the decreases in the Hb level from days 7 to 14 were probably due partly to natural fluctuations in the Hb level and variations in using the HemoCue machine. Overall, there was essentially no change in the mean Hb concentration between days 14 and 7, contrasting with the mean decrease of approximately 0.7 g/dL between days 0 and 7. Thus, the first week represents the highest risk for requiring a blood transfusion, as seen in our patient who was transfused on day 4.

Patients rapidly cleared their parasites and were protected by the long half-life of piperaquine from recurrent *P. vivax* or *P. falciparum* parasitemia, yet just over 20% had a poor recovery of the Hb level. Although counterintuitive, the factors associated with good recovery of the Hb level (ie, G6PD deficiency and a higher baseline parasitemia level) were similar to those associated with a greater initial decrease in the Hb level and an enhanced decrease in the uninfected RBC count. These changes trigger a bone marrow response that is able to overcome the suppressive effects of tumor necrosis factor α [41] and hemozoin [42] on the bone marrow RBC series. Thalassemia was not an important factor for the recovery of the Hb level, the total malaria-attributable change in the Hb level following treatment, or reticulocytosis, suggesting little physiological effect on erythropoiesis in *P.* vivax.

Our study had limitations. We recruited only 18 G6PD^−^ patients, among whom there was 1 predominant variant, and most patients were adults with Hb concentrations ≥ 8 g/dL, thus limiting the generalizability of our findings and statistical power. Moreover, multiple comparisons may have produced significant results by chance. We did not have a control group of patients who received dihydroartemisinin/piperaquine alone; this would have given us important dynamic data on Hb levels without the influence of PQ.

To conclude, thalassemic and highly parasitemic G6PD^−^ patients are at greatest risk of PQ_0.75_-induced acute hemolytic anemia and should be identified and followed up closely in the first week. A simple package of alerts for patients and health workers should be developed when malaria control programs rollout PQ and TQ. Larger studies from other malaria-endemic regions are needed to identify patients at risk of clinically significant acute hemolytic anemia, and mapping studies of G6PD deficiency and thalassemia would inform malaria control programs of potential areas of hemolytic risk. Alternative regimens not requiring monitoring should be explored [43].

## Supplementary Data

Supplementary materials are available at *The Journal of Infectious Diseases* online. Consisting of data provided by the authors to benefit the reader, the posted materials are not copyedited and are the sole responsibility of the authors, so questions or comments should be addressed to the corresponding author.

jiz313_suppl_Supplementary_Tabs_Figures

## Notes

Select data generated and analyzed during this study are included in this published article and the Supplementary Materials. Requests for additional data can be made in the first instance to the corresponding author, whose institution has a data access committee that considers requests for data.

***Acknowledgments.*** We thank the patients, for volunteering to join this study; the nurses and laboratory staff; the Drug Safety and Monitoring Board, for helpful advice; Drs Toby Leslie (chairperson), Chanthap Lon, and Isabella Ribeiro; and Prof Lucio Luzzatto.

S. Kheng (principal investigator), S. M., W. R. J. T., S. B., D. M., K. B., N. T., and E. C. developed the protocol. S. Kheng, S. M., W. R. J. T., N. T., K. K., P. S., S. Kim, C. V., P. L., J. K. B., and S. B. executed the study and provided training and supervision. C. M. C. contributed intellectually to the study. V. K., S. Kim, and K. K. managed the data. S. Kim, A. K., P. T., and D. M. performed laboratory analyses. W. R. J. T. and M. M. analyzed the data. W. R. J. T. and M. M. wrote the first draft of the manuscript. K. B., D. M., S. B., and A. P. critically reviewed the manuscript. All authors read the final manuscript.

***Financial support.*** This work was supported by the World Health Organization; Expertise France International, through the 5% Initiative (to W. R. J. T. as a consultant in operational research to the Cambodian National Center for Parasitology, Entomology, and Malaria Control); the Wellcome Trust (grant B9RJIXO to J. K. B.); and the French Ministry of Foreign Affairs (to D. M.).

***Potential conflicts of interest.*** All authors: No reported conflicts.
