# Corrigendum to: Hemolytic Dynamics of Weekly Primaquine Antirelapse Therapy Among Cambodians With Acute *Plasmodium vivax* Malaria With or Without Glucose-6-Phosphate Dehydrogenase Deficiency

**DOI:** 10.1093/infdis/jiz530

**Published:** 2019-11-26

**Authors:** Walter R J Taylor, Sim Kheng, Sinoun Muth, Pety Tor, Saorin Kim, Steven Bjorge, Narann Topps, Khem Kosal, Khon Sothea, Phum Souy, Chuor Meng Char, Chan Vanna, Po Ly, Virak Khieu, Eva Christophel, Alexandra Kerleguer, Antonella Pantaleo, Mavuto Mukaka, Didier Menard, J Kevin Baird

**Affiliations:** 1 National Center for Parasitology, Entomology, and Malaria Control, Phnom Penh; 2 Institut Pasteur du Cambodge, Phnom Penh; 3 World Health Organization (WHO) Cambodia Country Office, Phnom Penh; 4 Pailin Referral Hospital, Pailin; 5 Anlong Veng Referral Hospital, Anlong Venh; 6 Pramoy Health Center, Veal Veng, Cambodia; 7 Service de Médecine Tropicale et Humanitaire, Hôpitaux Universitaires de Genève, Switzerland; 8 Mahidol Oxford Tropical Medicine Research Unit, Bangkok, Thailand; 9 WHO Western Pacific Regional Office, Manila, the Philippines; 10 Department of Biomedical Science, University of Sassari, Italy; 11 Centre for Tropical Medicine, Nuffield Department of Medicine, University of Oxford, United Kingdom; 12 Malaria Genetics and Resistance Group, Biology of Host-Parasite Interactions Unit, Institut Pasteur, Paris, France; 13 Eijkman Oxford Clinical Research Unit, Eijkman Institute of Molecular Biology, Jakarta, Indonesia

In “Hemolytic Dynamics of Weekly Primaquine Antirelapse Therapy Among Cambodians With Acute Plasmodium vivax Malaria With or Without Glucose-6-Phosphate Dehydrogenase Deficiency” by Taylor et al [J Infect Dis 2019; 220(11): 1750–60], *P* values should be *P* < 0.001 where 0.000 is written. This has been updated in the article.

The authors regret these errors.

