# Supplementary material for: Hemolytic Dynamics of Weekly Primaquine Antirelapse Therapy Among Cambodians With Acute Plasmodium vivax Malaria With or Without Glucose-6-Phosphate Dehydrogenase Deficiency
Source: J Infect Dis. 2019 Sep 24;220(11):1750–60. doi: 10.1093/infdis/jiz313 (PMC6804333; doi:10.1093/infdis/jiz313)

Additional file 1. Calculating the red blood cell loss.

First, the blood volume (BV in liters) was estimated: 70 ml/kg (males), 65 ml/kg (females), 75 ml/kg (children both sexes).

The total number of circulating red blood cells, tRBC = BV x N of RBC/µL from the full blood count

The total number of lost RBCs = tRBC x fractional fall in hemoglobin, ΔHbF (100x (nadir Hb - D0Hb)/D0Hb

The total number of lost infected RBCs = tRBC x % parasitemia

Total number of lost uninfected RBCs = tRBC - iRBC

Additional file 2. Trial profile.

Additional file 3. Histogram showing the days of nadir hemoglobin.


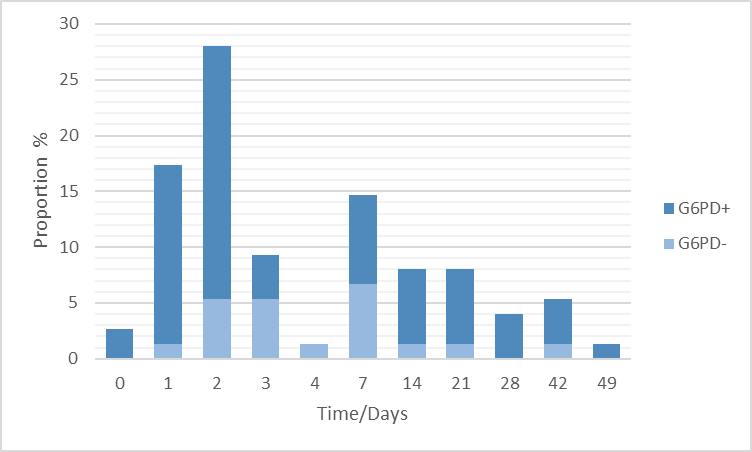


Additional file 4. Changes in hemoglobin and creatinine concentrations in 12 patients with clinically concerning hemoglobin declines.

| **Hemoglobin g/dL** | | | **Fractional fall** | **Serum creatinine µmol/L** | | | |  |  | **G6PD** | **Hemoglobin** |
| --- | --- | --- | --- | --- | --- | --- | --- | --- | --- | --- | --- |
| **D0** | **Nadir** | **Fall** | **%** | **D0** | **D7** | **D28** | **D56** | **Age** | **Sex** | **variant** | **type** |
| 14.8 | 9 | -5.8 | 39.19 | 47 | 47 | 33 | 36 | 16 | M | Viangchan | HbAE |
| 16 | 11 | -5 | 31.25 | 75 | 70 | 82 | - | 38 | M | Viangchan | Normal |
| 16.3 | 12.1 | -4.19 | 25.77 | 75 | 59 | 57 | 56 | 34 | M | Wild type | HbAE |
| 12.8 | 8.9 | -3.9 | 30.47 | 29 | 52 | 42 | 36 | 11 | M | Viangchan | Normal |
| 14.5 | 10.6 | -3.9 | 26.90 | 84 | 66 | 65 | 51 | 18 | M | Wild type | Normal |
| 14.4 | 10.9 | -3.5 | 24.31 | 67 | 73 | - | - | 32 | M | Wild type | HbAE |
| 14.8 | 11.3 | -3.5 | 23.65 | 74 | 66 | 68 | 72 | 30 | M | Wild type | Normal |
| 13.2 | 9.8 | -3.4 | 25.76 | 38 | 43 | 38 | 43 | 51 | F | Viangchan | Normal |
| 15.6 | 12.4 | -3.2 | 20.51 | 79 | 80 | 70 | 64 | 20 | M | Wild type | HbAE |
| 14.1 | 10.9 | -3.2 | 22.70 | 58 | 65 | 71 | 77 | 35 | M | Wild type | Normal |
| 13.9 | 10.7 | -3.2 | 23.02 | 77 | 56 | 58 | 76 | 22 | M | Viangchan | Normal |
| 10 | 7.2 | -2.8 | 28.00 | 53 | 52* | 62 | 64 | 23 | M | Viangchan | Normal |

HbAE: heterozygous hemoglobin E

* creatinine values on D3 & D5 were taken at the local hospital and were 70.7 & 79.6 µmol/L, respectively.

Additional file 5. Hemoglobin concentrations from baseline to D14 and D14-D7 hemoglobin differences in patients who had a decline in hemoglobin between D14 and D7.

| **Day 14-7**  **hemoglobin** | **Day 0** | **Day 1** | **Day 2** | **Day 3** | **Day 7** | **Day 14** | **G6PD**  **status** | **Hemoglobin type** | **Day of nadir hemoglobin** |
| --- | --- | --- | --- | --- | --- | --- | --- | --- | --- |
| -0.3 | 9.6 | 8.2 | 9.4 | 9 | 10 | 9.7 | 1 | HbAE | 1 |
| -1.2 | 14.6 | 12.1 | 13.1 | 13 | 13.7 | 12.5 | 0 | HbAE | 1 |
| -0.9 | 12.7 | 11.9 | 13.4 | 13.8 | 15.5 | 14.6 | 0 | Normal | 1 |
| -0.5 | 12.7 | 11.8 | 15.2 | 15.6 | 13.3 | 12.8 | 0 | Normal | 1 |
| -2.8 | 13 | 11.3 | 12.3 | 12.2 | 14.4 | 11.6 | 0 | HbAE | 1 |
| -1.1 | 11.3 | 10.2 | 9.2 | 10.3 | 11.3 | 10.2 | 0 | Normal | 2 |
| -1.3 | 14.3 | 14 | 13 | 14.1 | 14.8 | 13.5 | 0 | Normal | 2 |
| -0.5 | 12.5 | 12.1 | 11.8 | 12.3 | 12.6 | 12.1 | 0 | α thalassemia | 2 |
| -0.3 | 12 | 11.8 | 11.2 | 11.7 | 12.4 | 12.1 | 0 | Normal | 2 |
| -2.8 | 10.9 | 10 | 9.4 | 10.9 | 12.3 | 9.5 | 0 | β thalassemia | 2 |
| -1 | 14.8 | 14.4 | 13.8 | 15.4 | 15.4 | 14.4 | 0 | Normal | 2 |
| -1.8 | 12.2 | 11.2 | 10.7 | 11 | 13 | 11.2 | 1 | Normal | 2 |
| -1.3 | 15.6 | 12.5 | 12.4 | 13 | 14.9 | 13.6 | 0 | HbAE | 2 |
| -0.5 | 13.8 | 12.7 | 11.2 | 12.6 | 13.1 | 12.6 | 0 | Normal | 2 |
| -0.7 | 14.2 | 13.5 | 11.9 | 14.5 | 15.4 | 14.7 | 0 | Normal | 2 |
| -1.2 | 13 | 12.5 | 10.8 | 13.8 | 13.4 | 12.2 | 0 | HbAE | 2 |
| -0.3 | 13.2 | 11 | 9.9 | 9.8 | 10.5 | 10.2 | 1 | Normal | 3 |
| -2.2 | 16.3 | 14.4 | 14.2 | 14 | 14.3 | 12.1 | 0 | HbAE | 14 |
| -1 | 14.8 | 14.3 | 13.7 | 13.6 | 14.6 | 13.6 | 0 | Normal | 14 |
| -3.2 | 12.5 | 12.1 | 11.7 | 12.2 | 14.6 | 11.4 | 0 | HbAE | 14 |
| -0.8 | 15.6 | 15 | 14.9 | 15.6 | 15.3 | 14.5 | 0 | Normal | 14 |
| -1.6 | 12.6 | 10.1 | 10.7 | 10.8 | 11.7 | 10.1 | 1 | α thalassemia | 14 |
| -0.2 | 13.5 | 13.7 | 12.9 | 13.5 | 12.7 | 12.5 | 0 | β thalassemia | 14 |
| -0.6 | 13.1 | 12.5 | 10.9 | 10.6 | 11.5 | 10.9 | 0 | HbAE | 21 |
| -1.4 | 14.4 | 12.4 | 13.1 | 13.3 | 14.6 | 13.2 | 0 | HbAE | 21 |
| -0.2 | 14.5 | 13 | 12.8 | 12.7 | 13 | 12.8 | 0 | β thalassemia | 28 |
| -0.2 | 15.8 | 14.5 | 14.5 | 14.5 | 15.7 | 15.5 | 0 | Normal | 28 |
| -0.1 | 14.6 | 13.4 | 13.8 | 13.1 | 13 | 12.9 | 0 | HbAE | 42 |
| -0.9 | 14.5 | 12.4 | 13.1 | 13.5 | 12.9 | 12 | 0 | Normal | 42 |
| -0.7 | 14 | 14.7 | 14 | 13.7 | 14.3 | 13.6 | 0 | Normal | 49 |

G6PD status: 0-normal, 1=deficient

HbAE: heterozygous hemoglobin E

Additional file 6. Figure of changes in unconjugated bilirubin concentrations over time by G6PD status.

Additional file 7. Figure of changes in conjugated bilirubin concentrations over time by G6PD status.

Additional file 8. Urine Hillmen color scores in all patients during the course of the study.

|  | **D0** | **D3** | **D7** | **D14** | **D21** | **D28** | **D35** | **D42** | **D49** | **D56** |
| --- | --- | --- | --- | --- | --- | --- | --- | --- | --- | --- |
| *G6PD normal* |  |  |  |  |  |  |  |  |  |  |
| Minimum | 0 | 0 | 0 | 0 | 0 | 0 | 0 | 0 | 0 | 0 |
| Median | 1 | 1 | 1 | 1 | 1 | 1 | 1 | 1 | 1 | 1 |
| Maximum | 2 | 1 | 1 | 1 | 1 | 1 | 1 | 1 | 1 | 1 |
| *G6PD deficient* |  |  |  |  |  |  |  |  |  |  |
| Minimum | 0 | 0 | 1 | 0 | 0 | 0 | 0 | 0 | 0 | 0 |
| Median | 3 | 1 | 1 | 1 | 1 | 1 | 1 | 1 | 1 | 1 |
| Maximum | 4 | 2 | 2 | 2 | 3 | 1 | 3 | 1 | 2 | 1 |

Additional file 9. Urine Hillmen color score in the transfused G6PD deficient male patient each time he passed urine. Hemoglobin concentrations were 10, 8.8, 8.2, 7.5 and 7.2 g/dL on Days 0-4, inclusive.


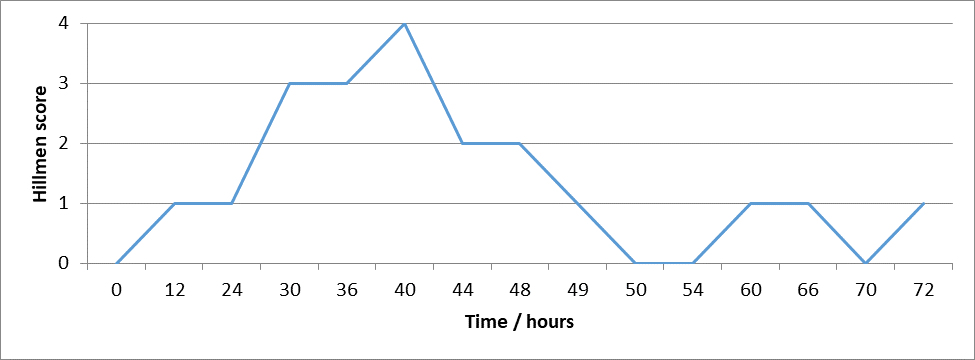

Supplement: jiz313_suppl_Supplementary_Tabs_Figures [file jiz313_suppl_supplementary_tabs_figures.doc]
